# Malnutrition is not related with emergence delirium in older patients after noncardiac surgery

**DOI:** 10.1186/s12877-021-02270-2

**Published:** 2021-05-17

**Authors:** Fang Zhang, Shu-Ting He, Yan Zhang, Dong-Liang Mu, Dong-Xin Wang

**Affiliations:** Department of Anesthesiology, Peking University First Hospital, Xishiku Street No.8, 100034 Beijing, China

**Keywords:** Malnutrition, Emergence delirium, Nutritional risk screening 2002, Older patient, Non-cardiac surgery

## Abstract

**Background:**

Delirium is one of the most common complications in older surgical patients. Although previous studies reported that preoperative malnutrition was related with postoperative delirium (POD), there was lack of evidence to illustrate the relationship between malnutrition and emergency delirium (ED). The objective of this study was to investigate the relationship between preoperative malnutrition and ED in older patients undergoing noncardiac surgery.

**Methods:**

The study was carried out in accordance with STROBE guidelines. This was a secondary analysis of a prospective cohort study. Older patients (65–90 years) who underwent noncardiac surgery under general anesthesia were enrolled in Peking University First Hospital.

**Results:**

915 patients were enrolled. The incidence of malnutrition was 53.6 % (490/915). The incidence of emergency delirium was 41.8 % (205/490) in malnutrition group and 31.5 % (134/425) in control group, P < 0.001. After adjusting confounding factors (i.e., age, cognitive impairment, American Society of Anesthesiologists classification (ASA), duration of surgery, pain score, low body temperature and allogeneic blood transfusion), malnutrition was not associated with increased risk of emergency delirium (OR = 1.055, 95 % CI 0.767–1.452, P = 0.742).

**Conclusions:**

Malnutrition was common in older patients undergoing non-cardiac surgery, but it’s not related with emergence delirium after adjusted for confounders.

**Trial registration:**

Chinese Clinical Trial Registry (http://www.chictr.org.cn) (ChiCTR-OOC-17,012,734).

**Supplementary Information:**

The online version contains supplementary material available at 10.1186/s12877-021-02270-2.

## Introduction

Malnutrition has been considered as a great challenge to patient’s safety in perioperative settings [1, 2]. It is estimated that 24–51 % of surgical patients are at risk of malnutrition [3, 4]. Its incidence reaches up to 60-86 % in the older patients [4, 5]. Malnutrition is associated with increased risk of morbidity (i.e., gastrointestinal fistula, wound dehiscence, and infection) and mortality [1–5].

Delirium is one of the most common complications in older surgical patients. It is manifested as acute disturbance of attention and cognitive function, abnormal sleep cycle and disorganized thinking [6]. According to its onset time, delirium can be divided into emergence delirium (ED, in the immediate post-anesthesia period) and postoperative delirium (POD, usually within postoperative 5 days) [7]. Nowadays, the adverse effect of ED on patient’s outcome has raised up major concern. ED is highly associated with increased risk of postoperative complications, such as increased risk of POD, prolonged length of in-hospital stay and increased risk of readmission [8–10]. The underlying mechanism of ED is multifactorial and still not clear. Common risk factors of ED in previous studies included frailty, comorbidities (i.e., cognitive impairment and diabetics), use of volatile anesthetics and benzodiazepines, higher pain intensity, and urinary catheterization [8, 11, 12].

Although previous studies reported that preoperative malnutrition was related with POD, there was lack of evidence to illustrate the relationship between malnutrition and ED. Two cohort studies reported that preoperative malnutrition was associated with increased risk of postoperative delirium in patients undergoing on pump coronary artery bypass grafting [13, 14]. The relationship between malnutrition and POD were also investigated in patients undergoing orthopedic surgery and other non-cardiac surgeries [15, 16]. However, no study reported the association between preoperative malnutrition and ED.

This study was designed to investigate the relationship between preoperative malnutrition and emergence delirium in older patients after non-cardiac surgery.

## Methods

### Study design

Present study was secondary analysis of a prospective observational study which was approved by the Clinical Research Ethics Committee of Peking University First Hospital on August 4, 2017 (2017[1419], Beijing, China) and registered with Chinese Clinical Trial Registry on September 19, 2017 (chictr. org.cn, ChiCTR-OOC-17,012,734) [9]. Written informed consent was obtained from all participants or their legal representatives.

### Participants

Older patients (aged 65–90 years) were included if they were scheduled to undergo noncardiac surgery with an expected duration ≥ 2 h under general anesthesia. Patients who met any of the following criteria were excluded: (1) refused to participate in the study; (2) previous history of schizophrenia, epilepsy, Parkinson’s Disease, or myasthenia gravis; (3) unable to communicate due to severe dementia, comatose or language barrier; (4) traumatic brain injury or neurosurgery; (5) an American Society of Anesthesiologists (ASA) classification of IV or above; or (6) Emergency surgery.

### Malnutrition

Preoperative malnutrition was defined as nutritional risk screening 2002 (NRS2002) ≥ 3. NRS 2002 contains two components: nutritional status and disease severity, giving a total score of 0–6 (Supplementary Table S1) [17]. If patient’s age is 70 years or above, an additional 1 score should be added to the above total score. Nutritional status was estimated by using BMI, percent of recent weight loss and change in food intake. Each item of undernutrition is classified into absent, mild, moderate, and severe with relevant score of 0–3 respectively. Disease severity is reflection of stress metabolism which is divided into normal to severe status with score 0–3.

### Emergence delirium

Emergence delirium was defined as any episode of delirium during PACU stay and was assessed by the Confusion Assessment Method for the Intensive Care Unit (CAM-ICU) at 10 and 30 min after PACU admission, and before PACU discharge [9, 18].

Before assessing delirium assessment, the level of sedation/agitation was evaluated with the Richmond Agitation Sedation Scale (RASS) [19]. If the patient was over-sedated or unarousable (-4 or -5 on the RASS), delirium assessment was stopped and the patient was marked as comatose. If the RASS was between − 3 and + 5, delirium assessment was performed. Emergence delirium was classified into 3 subtypes, i.e., hyperactive (with a consistently positive RASS, from + 1 to + 4), hypoactive (with consistently neutral or negative RASS, from 0 to − 3) and mixed. During the study period, investigators who performed delirium assessment did not participate in perioperative care of the enrolled patients.

All investigators who were in charge of delirium assessment were trained to use the CAM-ICU by a psychiatrist [20]. Confusion Assessment Method (CAM) were also trained for postoperative delirium assessment at the meantime [21]. The training program included lectures introducing delirium and the CAM/CAM-ICU, as well as simulation courses with patient-actors [9]. The initial training continued until the diagnosis of delirium reached 100 % agreement with the psychiatrist. The training process was repeated at least two times a year. Investigators who performed delirium assessment did not participate in perioperative care of the enrolled patients.

### Anesthesia and perioperative management

All patients received standard monitoring on arrival in the operating room including electrocardiogram, non-invasive blood pressure, pulse oxygen saturation, and urine output. During general anesthesia, end-tidal carbon dioxide and bispectral index (BIS) were monitored. Invasive arterial pressure and central venous pressure were used if considered as necessary.

Anesthesia induction was completed by propofol and/or etomidate, opioids (sufentanil and/or remifentanil) and muscle relaxants (rocuronium or cisatracurium). Anesthesia maintenance was conducted by infusion of propofol and/or sevoflurane inhalation. Nitrous oxide could be used as supplementary in necessary. Opioids and muscle relaxants were administered when considered necessary. The target was to maintain bispectral index between 40 and 60.

Muscle relaxants were stopped for at least 30 min before the end of surgery; propofol infusion and/or sevoflurane inhalation were decreased or stopped according to BIS monitoring; sufentanil was administered in necessary. At the end of surgery, Neostigmine (0.05 mg/kg) and atropine (0.02 mg/kg) were used to reverse residual effect of neuromuscular blockade. Patients were extubated when they met the following criteria: (1) easy to wake up; (2) sufficient reflexes that protect the airway; (3) adequate gas exchange (respiration rate 10–30 breaths per minute and tidal volume > 6 ml/kg); and (4) acceptable hemodynamic status (systolic blood pressure ≥ 90 mmHg and heart rate ≤ 100 beats per minute).

As a routine practice, patients were transferred to the PACU after extubation. Patients were monitored in PACU for at least 30 min and then transferred to the general ward when the Aldrete score was higher than 9. Pain severity was assessed with the numeric rating scale (NRS, an 11-point scale where 0 = no pain and 10 = the worst pain). Moderate to severe pain (NRS pain score > 3) was managed with intravenous opioids and/or non-steroid anti-inflammatory drugs (NSAIDs). Tympanic temperature was measured with an infrared ear thermometer. Patients with hypothermia (< 36 °C) were managed with warm air blanket.

### Outcome

Primary outcome was to investigate the relationship between malnutrition and occurrence of emergence delirium.

### Data collection and postoperative follow-up

Baseline data included demographics, education, diagnosis, comorbidities, smoking, alcoholism, Charlson Comorbidity Index [22], and ASA classification. Baseline cognitive function was evaluated with the Mini-Mental State Examination (MMSE, scores range from 0 to 30, with higher scores indicating better function) at one day before surgery. Cognitive impairment was defined as MMSE < 27. Intraoperative data included types of anesthetic drugs, site of surgery, as well as duration of surgery.

Postoperative data during PACU stay included NRS pain score, tympanic temperature, and length of PACU stay. In the general wards, patients were followed up twice daily until the 5th day after surgery for the occurrence of delirium and non-delirium complications. From the 6th day after surgery, patients were followed up weekly until discharge. For those who were discharged from the hospital, follow-ups were performed by telephone interview.

### Sample size

As a secondary analysis, we calculated the statistical power based on available data. The incidence of ED was 41.8 % (205/490) in malnutrition group and 31.5 % (134/425) in control group. Assuming significance at 0.05, this would yield a power of 0.90.

### Statistical analysis

Normality of continuous data was tested by Kolmogorov-Smirnov method in prior. Data with normal distribution were presented as mean ± standard deviations (SD) and differences between groups were compared by independent t sample. Data without normal distribution were presented as median (IQR) and differences between groups were compared by Mann-Whitney U test. Categorical data was presented by number (percentage) and differences between groups were compared by Chi-square test.

The relationship between malnutrition and ED was firstly analyzed by univariate analysis, followed by multivariable logistic regression analysis adjusted for confounding factors including the baseline characteristics and perioperative variables that showed an imbalance between patients with and without ED (i.e., P value < 0.05).

Two-sided P < 0.05 was considered as statistical significance. Statistical analysis was performed using SPSS 24 Inc. Chicago, IL, USA.

## Results

### Participants

**Fig. 1:**
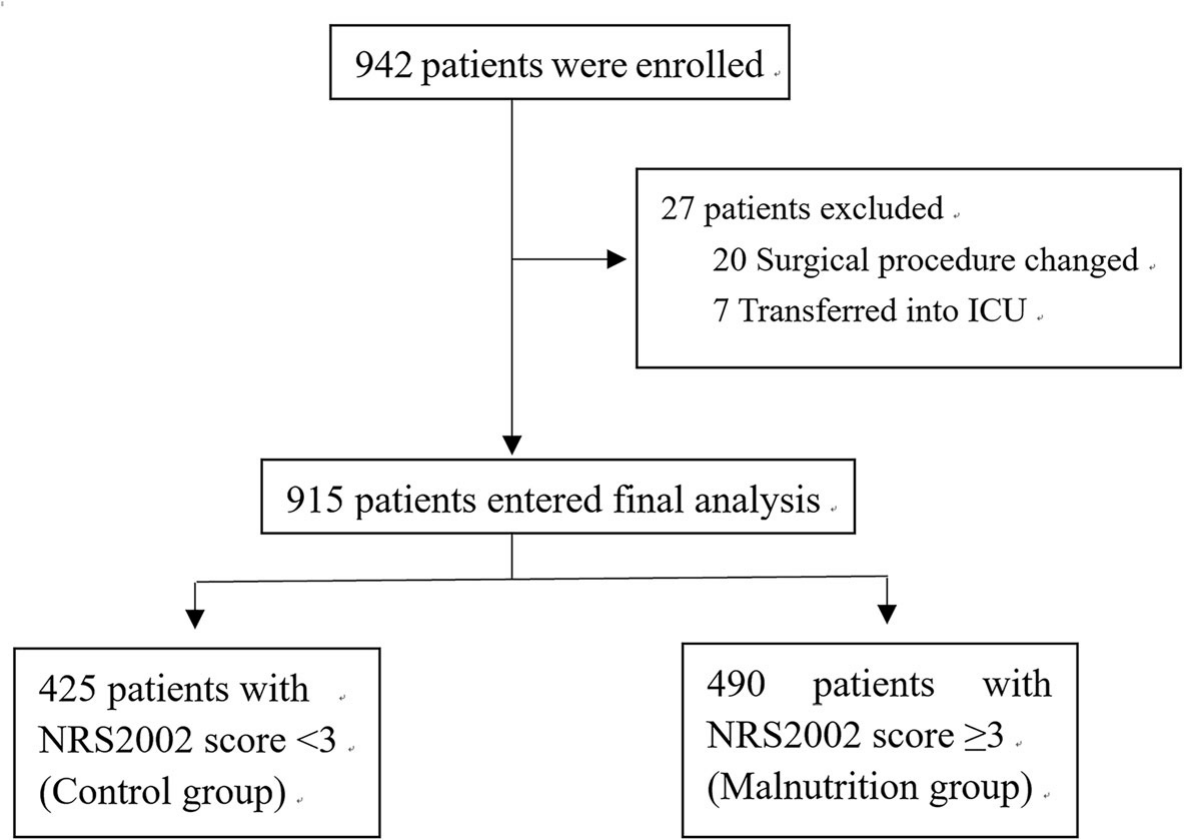
Flowchart

From September 21, 2017 to April 10, 2019, 984 patients met with inclusion criteria. Finally, 915 patients were included with mean age of 71.6 ± 5.2 years, Fig. 1.

The mean age of patients with malnutrition was higher than that of patients in control group (73.6 ± 5.2 vs. 69.3 ± 4.2, P < 0.001), Table 1. In comparison with control group, patients in malnutrition group had lower body mass index and serum albumin level, but higher incidence of preoperative comorbidities (i.e., stroke and Malignant tumor), all P values < 0.05, Table 1. The percentage of abdominal surgery was higher in malnutrition group than control group (P < 0.001), Table 2.

**Table 1:** Baseline variables

Variables	All patients (n = 915)	Control group (n = 425)	Malnutrition group (n = 490)	P
Age, year, mean ± SD	71.6 ± 5.2	69.3 ± 4.2	73.6 ± 5.2	< 0.001
≥75 years, n (%)	264 (28.9 %)	61(14.4 %)	203(41.1 %)	< 0.001
Male, n (%)	548 (59.9 %)	180(44.0 %)	310(56.0 %)	0.025
BMI, kg/m^2^, mean ± SD	24.2 ± 3.5	25.2 ± 3.0	23.2 ± 3.6	< 0.001
Preoperative comorbidity, n (%)				
Stroke	52 (5.7 %)	14(3.3 %)	38(7.8 %)	0.004
Hypertension	475 (51.9 %)	226(53.2 %)	249(50.8 %)	0.476
Coronary heart disease	129 (14.1 %)	61(14.4 %)	68(13.9 %)	0.837
Arrhythmia	57 (6.2 %)	26(6.1 %)	31(6.3 %)	0.896
Pulmonary disease ^a^	66 (7.2 %)	27(6.4 %)	39(8.0 %)	0.349
Diabetes	219 (23.9 %)	108(25.4 %)	111(22.7 %)	0.329
Hyperlipidemia	95 (10.4 %)	49(11.5 %)	46(9.4 %)	0.290
Hepatic dysfunction ^b^	45 (4.9 %)	14(3.3 %)	31(6.3 %)	0.034
Malignant tumor ^c^	105 (11.5 %)	37(8.7 %)	68(13.9 %)	0.014
Chronic smoking ^d^	223 (24.4 %)	105(24.7 %)	118(24.1 %)	0.826
Cognitive impairment ^e^	597 (65.2 %)	260(61.2 %)	337(68.8 %)	0.016
History of surgery, n (%)	491(53.7 %)	221(52.0 %)	270(55.1 %)	0.881
CCI, median (IQR)^e^	2 (2,3)	2(2,3)	3(2,3)	< 0.001
ASA classification, n (%)				0.001
Class II	678 (74.0 %)	336(79.1 %)	342(69.8 %)	
Class III	237 (26.0 %)	89(20.9 %)	148(30.2 %)	
Laboratory tests,				
Albumin, g/L, mean ± SD	40.6 ± 4.7	41.7 ± 3.3	39.6 ± 5.5	< 0.001
<30 g/L, n (%)	22(2.4 %)	0(0.0 %)	22(4.5 %)	< 0.001
Creatinine, µmol/L, mean ± SD	80.2 ± 20.9	77.4 ± 18.7	82.5 ± 22.3	0.004
Glucose, mmol/L, mean ± SD	6.0 ± 1.7	6.0 ± 1.6	6.1 ± 1.8	0.332
Hematocrit, %, mean ± SD	39.4 ± 5.3	40.8 ± 4.7	38.2 ± 5.5	0.002
Barthel index, median (IQR) ^f^	100 (100,100)	100 (95,100)	100 (100,100)	0.606
NRS 2002, median (IQR)	3 (2,4)	2 (1,2)	3 (3,5)	< 0.001

*SD *standard deviation, *BMI *body mass index, *CCI *Charlson Comorbidity Index, *IQR *interquartile range, *ASA *American Society of Anesthesiology, *NRS 2002 *nutritional risk screening 2002

^a^ Pulmonary disease included chronic obstructive pulmonary disease and asthma

^b^ Hepatic dysfunction was defined as Alanine transaminase and/or aspartate transaminase higher than 5 times of the upper normal limit

^c^ Malignant tumor was defined as carcinoma (carcinoma, squamous cell carcinoma and adenocarcinoma), sarcoma and undifferentiated carcinoma

^d^ Chronic smoking was defined as half a pack of cigarettes per day for at least 2 years

^e^ Cognitive impairment was defined as Mini-Mental State Examination (MMSE) score less than 27

^f^ Barthel index was used to assess activity of daily life (0-100 score, and higher score indicates better activity)

**Table 2:** Perioperative variables

Variable	All patients (n = 915)	Control group (n = 425)	Malnutrition group (n = 490)	P
Type of anesthesia, n (%)				< 0.001
General anesthesia	420 (45.9 %)	226(53.2 %)	194(39.6 %)	
General-PNB anesthesia ^a^	495 (54.1 %)	199(46.8 %)	296(60.4 %)	
Duration of surgery, h, mean ± SD	3.4 ± 1.2	3.3 ± 1.1	3.4 ± 1.2	0.154
Location of surgery, n (%)				< 0.001
Intra-thoracic	198 (21.6 %)	135(31.8 %)	63(12.9 %)	
Intra-abdominal	530 (57.9 %)	142(33.4 %)	388(79.2 %)	
Spinal/extremities/others	187 (20.4 %)	148(34.8 %)	39(8.0 %)	
Estimated blood loss, ml, median (IQR)	100 (10, 250)	100(10,300)	100(10,200)	0.061
Allogeneic blood transfusion, n (%)	79 (8.6 %)	42(9.9 %)	37(7.6 %)	0.210
Total fluid infusion, ml, mean ± SD	2200 (1600, 2850)	2100(1600,2600)	2350(1800,3100)	< 0.001
Urine output, ml, mean ± SD	400 (250, 600)	400(250,650)	400(250,600)	0.564
Temperature at PACU admission,(°C)	36.1 ± 0.4	36.2 ± 0.4	36.1 ± 0.4	0.844
T < 36 °C, n (%)	301(32.9 %)	119(28.0 %)	182(37.1 %)	0.003
Average NRS pain score in PACU, median (IQR)	2.0 (1.3,3.0)	2.0(1.0,3.3)	2.0(1.3,3.0)	0.695
LOS in PACU, min, mean ± SD	42.7 ± 15.0	42.9 ± 15.0	42.5 ± 15.0	0.807
Postoperative LOS in hospital, day, median (IQR)	8 (6,11)	7(5,9)	9(6,12)	< 0.001

*PNB *peripheral nerve block, *SD *standard deviation, *IQR *interquartile range, *PACU *post-anesthesia care unit, *NRS *numeric rating score, *LOS *length of stay

^a^ General-PNB anesthesia indicated that patients received both general anesthesia and peripheral nerve block (including epidural anesthesia)

### Malnutrition

According to NRS 2002, the incidence of malnutrition was about 53.6 % (490/915) in elderly patients undergoing non-cardiac surgery. Median score of NRS 2002 in malnutrition group was 3 (3,5) whereas 2 (1,2) in control group (P < 0.001).

### Emergence delirium

**Fig. 2:**
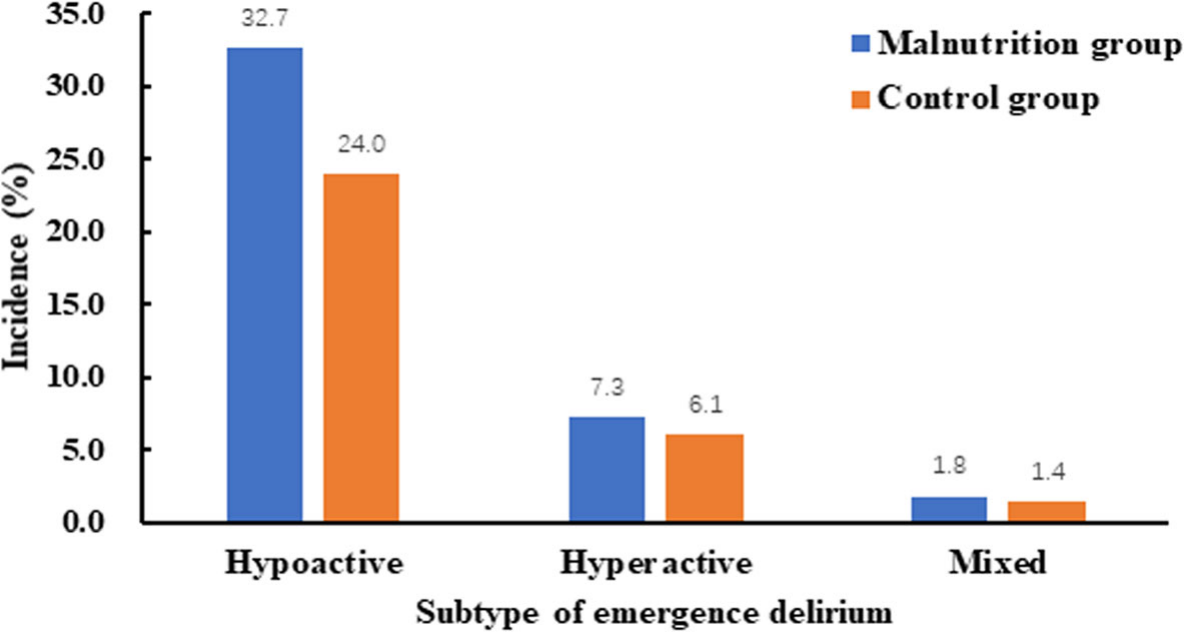
The percentage of subtype of emergency delirium and its subtype in two groups

The incidence of ED in malnutrition group was 41.8 % (205/490) which was higher than 31.5 % (134/425) in control group (P = 0.001), Fig. 2. The percentage of subtype of ED was also presented in Fig. 2.

### The relationship between malnutrition and emergence delirium

In univariate analysis, malnutrition was related with increased risk of ED (OR = 1.562, 95 %, CI 1.190–2.051, P = 0.001), Table 3. After adjusting confounding factors (i.e., age, cognitive impairment, ASA classification, duration of surgery, pain score, low body temperature and allogeneic blood transfusion), malnutrition was not related with ED (OR = 1.055, 95 % CI 0.767–1.452, P = 0.742), Table 3.

**Table 3:** The relationship between malnutrition and emergence delirium

Variables	Univariate analysis			Multivariable analysis		
	**OR**	**95 % CI**	**P**	**OR**	**95 % CI**	**P**
Age (per year increase)	1.077	1.049–1.105	< 0.001	1.079	1.050–1.110	< 0.001
Cognitive impairment (yes) ^a^	2.126	1.578–2.864	< 0.001	2.093	1.526–2.870	< 0.001
ASA classification (per grade increase)	1.440	1.065–1.946	0.018	1.092	0.782–1.524	0.606
Malnutrition (yes)^b^	1.562	1.190–2.051	0.001	1.055	0.767–1.452	0.742
Duration of surgery (per hour increase)	1.107	0.989–1.239	0.078	1.130	0.998–1.279	0.054
Allogeneic blood transfusion (yes)	2.179	1.369–3.469	0.001	1.782	1.076–2.952	0.025
Temperature < 36 °C at PACU admission (yes)	2.429	1.828–3.228	< 0.001	2.558	1.893–3.456	< 0.001
Average NRS pain score in PCAU (per score increase)	1.257	1.148–1.377	< 0.001	1.242	1.128–1.368	< 0.001

*ASA *American Society of Anesthesiologists, *PACU *post-anesthesia care unit, *NRS *numeric rating score, *OR *odds ratio, *CI *confidence interval

^a^ Cognitive impairment was defined as Mini-Mental State Examination (MMSE) score less than 27

^b^ Malnutrition was defined as NRS2002 score ≥ 3

## Discussion

Present study found that malnutrition was common in elderly patients undergoing non-cardiac surgery but it’s not associated with occurrence of emergence delirium after adjusted for confounders.

Perioperative malnutrition has raised up more concern because it’s highly related with poor patient’s outcome [1, 2, 23]. Nutritional risk screening 2002 (NRS 2002) is an assessment tool which has been widely validated in perioperative settings, it was developed by the Danish Association of Parenteral and Enteral Nutrition (DAPEN), and was recommended by European Society for Clinical Nutrition and Metabolism (ESPEN) [17]. In present study, the incidence of malnutrition is 53.6 % assessed by NRS2002 which was in line with previous studies [24–28].

CAM-ICU was employed to diagnose ED in present study. One strength of this study was that we assessed the phenotype of ED [29]. It seemed that the incidence of hypoactive ED was higher in patients with malnutrition than control group (32.7 % vs. 24.0 %, P = 0.014).

Although previous studies had reported the association between malnutrition and postoperative delirium, this study did not find the relationship between malnutrition and ED [13, 14, 30, 31]. Potential reasons might be attributed to the following three items. First, clinical manifestation of malnutrition varies greatly in surgical patients and a single assessment instrument is not sufficient to evaluate the nutrition status thoroughly. For example, several terms (i.e., sarcopenia, cachexia, and myostetosis) have been advocated to describe the different characteristics of malnutrition phenotypes and nutritional syndromes in malnourished patient [23]. But most assessment instruments merely focused on main characteristics of malnutrition such as body weight loss, decrement of oral intake and hypoalbuminemia [1, 2, 23]. It deserves to investigate if a multi-dimensional assessment instrument will provide better diagnostic performance. Second, microelement insufficiency might contribute to the occurrence of delirium but it’s not involved in the assessment instrument. Previous studies showed that microelement (i.e., magnesium and vitamin D) insufficiency was common in surgical patients and highly related with increased risk of delirium and postoperative cognitive dysfunction [32, 33]. Third, ED and POD share many common predisposing risk factors (such as aging, cognitive impairment, and frailty) but they also have different precipitating factors [8, 11, 12, 34, 35]. ED happens in the immediate post-anesthesia period and mainly caused by hypoxia, pain and the residual effect of general anesthetics [9, 36]. POD happens within postoperative 5 days and is mainly secondary to other complications such stroke, sepsis and respiratory failure [37]. Malnutrition was considered as the underlying risk factor of stroke, sepsis and respiratory failure in surgical patients [1–5]. One potential mechanism was that the effect of malnutrition on POD was mediated by the occurrence of other complications.

Beyond our result, the association between malnutrition and ED and POD should be investigated by further studies. First, metabolic abnormalities (such as ω-fatty acids insufficiency and glutamate–glutamine cycle dysfunction) possibly increase the vulnerability of the brain [38]. The vulnerable brain is at increased risk of ED and POD. Second, nutrients deficiency or excess are highly related with brain dysfunction and increase the risk of delirium [39]. It’s reported that correction of nutrient deficiency might decrease the risk of delirium in patients after cardiac surgery [40]. Third, multimodal prehabilitation had been proposed to reduce delirium and nutritional intervention was considered as an key step in the multidiscipline protocol [41].

Multivariable analysis showed that age, cognitive impairment, allogeneic blood transfusion, temperature < 36 °C at PACU admission and average NRS pain score in PCAU were related with increased risk of ED. This finding was in accordance with previous studies [8, 11, 12, 34, 35].

The strengths of present study included a relatively large sample size and strictly assessed delirium. This study had two limitations. First, our result was generated from a single center study which might limit its generality. Second, this was a cohort study which could not answer the causal relationship between malnutrition and delirium. Third, we did not collect the information of long-term medications. Previous studies showed that medications (i.e., statins) might be associated with delirium [42]. As many older patients have comorbidities and need long-term treatment, it would be interesting to investigate if these medications are related with ED.

## Conclusions

Our result showed that malnutrition was common in older patients undergoing non-cardiac surgery. The incidence of emergence delirium was higher in patients with malnutrition than control group. However, malnutrition was not related with emergence delirium after adjusted for confounders. Further studies are needed to elucidate the relationship between malnutrition and emergence delirium.

## Supplementary Information

Additional file 1: **Table S1. **Nutritional risk screening 2002 scale (NRS-2002).

## Data Availability

The datasets used and/or analyzed during the current study are available from the corresponding author on reasonable request.

## Abbreviations

POD: Postoperative Delirium
ED: Emergency Delirium
NRS 2002: Nutritional Risk Screening 2002
CAM-ICU: Confusion Assessment Method for the Intensive Care Unit
PACU: Post-Anesthesia Care Unit
ASA: American Society of Anesthesiologists
RASS: Richmond Agitation Sedation Scale
CAM: Confusion Assessment Method
BIS: Bispectral Index
NRS: Numeric Rating Scale
NSAIDs: Non-Steroid Anti-Inflammatory Drugs
CCI: Charlson Comorbidity Index
MMSE: Mini-Mental State Examination
DAPEN: Danish Association of Parenteral and Enteral Nutrition
ESPEN: European Society for Clinical Nutrition and Metabolism
