# Supplementary material for: Malnutrition is not related with emergence delirium in older patients after noncardiac surgery
Source: BMC Geriatr. 2021 May 17;21:319. doi: 10.1186/s12877-021-02270-2 (PMC8130292; doi:10.1186/s12877-021-02270-2)
Supplement: Supplementary file 1 — Table S1. Nutritional risk screening 2002 scale (NRS-2002). [file 12877_2021_2270_MOESM1_ESM.docx]

**Supplementary Table S1 Nutritional risk screening 2002 scale (NRS-2002)**

| **Nutritional status** | | **Severity of diseases** | |
| --- | --- | --- | --- |
| Absent Score 0 | Normal nutritional status | Absent Score 0 | Normal nutritional requirements |
|  |  |  |  |
| Mild Score 1 | Wight loss >5 % in 3 months  or  Food intake below 50–75% of normal requirement in preceding week | Mild Score 1 | Hip fractured  Chronic patients, in particular with acute complications: cirrhosis  COPD  Chronic hemodialysis, diabetes, oncology |
|  |  |  |  |
| Moderate Score 2 | Weight loss >5 % in 1 month (>15 % in 3 months)  or  BMI 18.5-20.5 + impaired general condition  or  Food intake 25-60 % of normal requirement in preceding week | Moderate Score 2 | Major abdominal surgery. Stroke.  Severe pnemonia, hematologic maligancy |
|  |  |  |  |
| Severe Score 3 | Weight loss >5 % in 1 month (>15 % in 3 months)  Or  BMI 18.5-20.5 + impaired general condition  or  Food intake 25-60 % of normal requirement in preceding week | Severe Score 3 | Head injury.  Bone marrow transplantation.  Intensive care patients (APACHE >10). |
| Age | if ≥70 years: add 1 to total score above |  |  |
